# Macrophage reprogramming by the ultraviolet filter benzophenone-1 promotes malignant phenotypes in breast cancer cells: integrated computational and experimental evidence

**DOI:** 10.3389/fmolb.2026.1946836

**Published:** 2026-10-13

**Authors:** Binglu He, Longying Ding, Xiaolu Yang, Tianqi Zhang, Li Ma

**Affiliations:** 1 Department of Breast Disease Center, The Fourth Affiliated Hospital of Hebei Medical University, Shijiazhuang, China; 2 Hebei Medical University, Shijiazhuang, China; 3 College of Veterinary Medicine, Shandong Agricultural University, Tai’an, China

**Keywords:** benzophe, none-1 (BP-1), breast cancer, macrophage, PLAUR, ultraviolet filters

## Abstract

**Background:**

Benzophenone-1 (BP-1) is a benzophenone ultraviolet absorber used in cosmetic and personal care products. Although BP-1 exhibits estrogenic activity, its broader effects in breast cancer remain unclear. This study investigated the molecular and cellular responses associated with BP-1 exposure.

**Methods:**

Integrated computational analyses were used to identify core targets and determine their cellular distribution. Molecular modeling evaluated BP-1–target binding. Macrophage polarization and secretion were assessed by immunofluorescence, RT-qPCR, enzyme-linked immunosorbent assay, and western blotting. The effects of macrophage-conditioned medium on breast cancer cells were examined using proliferation, migration, invasion, and epithelial-mesenchymal transition assays.

**Results:**

*PLAUR* was identified as the core BP-1-related candidate and was prominently expressed in macrophages. BP-1 increased expression of uPAR, the protein encoded by *PLAUR*, and shifted macrophages toward an M2-like phenotype. Conditioned medium from these macrophages enhanced malignant phenotypes in breast cancer cells.

**Conclusion:**

This study identifies a macrophage-associated response to BP-1 exposure that may contribute to malignant phenotypes in estrogen receptor-positive breast cancer, with *PLAUR*/uPAR as a candidate mediator. These findings provide a basis for further mechanistic and *in vivo* studies.

## 1 Introduction

Ultraviolet (UV) radiation is a persistent environmental risk factor for human health. Although global actions such as the Montreal Protocol have contributed to the recovery of stratospheric ozone, surface UV irradiance continues to vary with regional ozone changes, climate-related factors, season, and latitude, while individual UV exposure is further shaped by outdoor behavior (Solomon et al., 2016; Chubarova et al., 2020; Cahoon et al., 2013; Kimlin et al., 2019). Excessive UV exposure is closely associated with sunburn, photoaging, photodermatoses, and skin cancer, underscoring the continued need for effective photoprotection (Stump et al., 2023; Hughes et al., 2013; Green et al., 2011). In this context, UV filters have been widely incorporated into cosmetic and personal care products for photoprotection and formulation photostability (Lu et al., 2018; Li and Kannan, 2022). Benzophenone-type UV filters are an important class of organic UV absorbers. Among them, benzophenone-1 (BP-1), also known as 2,4-dihydroxybenzophenone, has been reported as a UV absorber and photostabilizer in cosmetic and personal care product formulations (Molina-Molina et al., 2008).

Because BP-1-containing cosmetic products may be applied directly to the skin and can remain on the skin for extended periods when used in leave-on formulations, dermal uptake represents an exposure route warranting consideration (Scientific Committee on Consumer Safety, 2024). BP-1 is a relatively small molecule with a molecular weight of 214.22 g/mol and moderate lipophilicity. Based on its physicochemical parameters, the safety assessment by the Scientific Committee on Consumer Safety (SCCS) reports that BP-1 is expected to have a low to moderate potential for dermal absorption (Scientific Committee on Consumer Safety, 2024). A human biomonitoring study of paired maternal and fetal samples detected BP-1 in maternal serum, amniotic fluid, and fetal and cord blood samples (Krause et al., 2018).

Structurally, BP-1 is a hydroxylated benzophenone derivative, characterized by hydroxyl substitution on the aromatic rings of the benzophenone scaffold (Molina-Molina et al., 2008; Suzuki et al., 2005). Because phenolic hydroxyl groups and aromatic ring structures may contribute to interactions with hormone-responsive systems, the potential endocrine-disrupting effects of hydroxylated benzophenone derivatives have attracted toxicological attention (Kerdivel et al., 2013). Previous structure-activity and comparative toxicological studies have shown that the position and number of hydroxyl substituents can influence the estrogenic activity of benzophenone derivatives and that some hydroxylated benzophenones may also exhibit anti-androgenic activity. Consistent with this structural and toxicological background, BP-1 has been reported to induce estrogen-like responses in estrogen receptor-positive breast cancer models, including changes in estrogen-responsive transcription, cell proliferation, cell cycle-related gene expression, and metastasis-associated gene expression (In et al., 2015). Beyond endocrine disruption, BP-1 has also been associated with intracellular reactive oxygen species (ROS) accumulation, impaired antioxidant defenses, and mitochondrial and nuclear injury. These changes may increase the likelihood of DNA damage and genomic instability (Tian et al., 2025; Amar et al., 2015). These observations suggest that BP-1 should not be regarded merely as an inert UV absorber or photostabilizer. Rather, its endocrine-disrupting activity, together with its potential oxidative stress and genotoxic effects, provides a biological rationale for further evaluating its effects in breast cancer.

Breast cancer is one of the most common malignancies among women worldwide and remains a major cause of cancer-related morbidity and mortality (Bray et al., 2024). Estrogen receptor (ER)-positive breast cancer represents the predominant molecular subtype of breast cancer. Although endocrine therapy has substantially improved the clinical management of estrogen receptor-positive breast cancer, tumor recurrence, metastatic progression, and therapeutic resistance remain important clinical challenges (Davies et al., 2013). Given the endocrine-active properties of BP-1, estrogen receptor-positive breast cancer provides a biologically relevant context for evaluating its potential biological effects.

Nevertheless, current evidence regarding BP-1 and breast cancer remains largely centered on classical endocrine receptor-dependent responses and limited tumor cell-intrinsic phenotypes. Breast cancer progression involves multiple interconnected processes, including tumor cell plasticity, epithelial-mesenchymal transition (EMT), invasion-associated regulatory networks, tumor microenvironment remodeling, and therapeutic response heterogeneity (Mani et al., 2008; Karnoub et al., 2007). Whether BP-1 participates in these broader biological processes, and through which molecular networks it may influence breast cancer progression, remains insufficiently understood. Therefore, a systematic and multi-level investigation is needed to clarify the potential mechanisms linking BP-1 exposure to breast cancer progression.

To address this gap, we integrated network toxicology, transcriptomic analysis, single-cell RNA sequencing analysis, virtual gene perturbation, molecular docking, and *in vitro* validation to investigate the potential role of BP-1 in breast cancer progression. This workflow was designed to identify candidate molecular mediators, evaluate their cellular distribution within the breast cancer microenvironment, and explore their potential involvement in tumor cell-microenvironment interactions and malignant phenotypes.

## 2 Materials and methods

### 2.1 Transcriptomic data acquisition and differential expression analysis

Three breast cancer transcriptomic datasets (GSE42568, GSE45827, and GSE70905) were downloaded from the Gene Expression Omnibus (GEO) database (https://www.ncbi.nlm.nih.gov/geo/), merged, and corrected for batch effects using the sva (Surrogate Variable Analysis) R package (Leek et al., 2012). Correction efficacy was assessed by boxplots and principal component analysis (PCA). Differential expression analysis between breast cancer and normal tissues was performed using the limma package. P values were adjusted for multiple testing using the Benjamini–Hochberg (BH) method to control the false discovery rate (FDR) (Reiner et al., 2003). Differentially expressed genes (DEGs) were defined as genes with an adjusted P value <0.05 and |log_2_ fold change| > 0.585. The results were visualized using heatmaps and volcano plots.

### 2.2 Weighted gene co-expression network analysis

To identify co-expression gene modules associated with breast cancer, a weighted gene co-expression network was constructed using the weighted gene co-expression network analysis (WGCNA) package in R based on the merged and normalized expression matrix. After removing genes with low variability, soft-thresholding powers were evaluated using the scale-free topology fit index (R^2^), which measures how closely the network connectivity distribution conforms to a scale-free topology (Zhang and Horvath, 2005). A soft-thresholding power with an R^2^ value greater than 0.9 was selected. Gene modules were identified using the dynamic tree-cutting algorithm. Module eigengenes were correlated with breast cancer and control phenotypes to screen for modules associated with breast cancer, and genes from these modules were combined with differentially expressed genes via union. The resulting gene set was defined as breast cancer-related genes and used for subsequent analyses.

### 2.3 Chemical characterization and toxicological target prediction of BP-1

The chemical information and canonical SMILES of BP-1 were retrieved from the PubChem database (https://pubchem.ncbi.nlm.nih.gov/). Its two-dimensional and three-dimensional molecular structures were also obtained from PubChem, and the three-dimensional structure was visualized using PyMOL software (https://www.pymol.org/). The molecular properties of BP-1 were obtained from ProTox 3.0 (https://tox.charite.de/). ProTox 3.0 was also used to predict toxicological targets and metabolic activities of BP-1.

### 2.4 Prediction of BP-1-related targets

To collect potential targets of BP-1, we used three databases for target prediction: ChEMBL (https://www.ebi.ac.uk/chembl/), the Similarity Ensemble Approach (SEA, https://sea.bkslab.org), and SwissTargetPrediction (http://www.swisstargetprediction.ch/). The union of target sets obtained from each database was visualized using a Venn diagram.

### 2.5 Identification and enrichment analysis of BP-1-related breast cancer candidate genes

BP-1-related targets from ChEMBL, SEA, and SwissTargetPrediction were integrated and duplicates were removed. These targets were then intersected with the breast cancer-related gene set obtained in Section 2.2 to identify candidate genes potentially linking BP-1 to breast cancer. The overlapping genes were subjected to downstream functional enrichment analysis.

Functional enrichment analysis of the candidate genes was performed based on Gene Ontology (GO), Kyoto Encyclopedia of Genes and Genomes (KEGG), and Reactome pathway annotations. GO analysis included biological process, cellular component, and molecular function categories. Enriched terms with *P* < 0.05 were considered significant.

### 2.6 Machine learning-based core gene selection

To identify core genes among the 15 candidate genes, we applied four machine learning methods for feature selection: least absolute shrinkage and selection operator (LASSO) regression, support vector machine recursive feature elimination (SVM-RFE), XGBoost, and Boruta. LASSO regression applies an L1 penalty to the feature coefficients. This shrinks the coefficients of unimportant genes toward zero (Tibshirani, 1996). We used 10-fold cross validation to find the optimal penalty parameter λ. Genes with nonzero coefficients were retained (Friedman et al., 2010). SVM-RFE recursively ranks and removes genes that contribute least to the SVM classification. The optimal feature subset was selected based on cross-validation error and accuracy (Guyon et al., 2002). XGBoost builds an ensemble of gradient boosted decision trees. It ranks gene importance using the gain metric. The top five genes were retained (Chen and Guestrin, 2016). We also used Shapley additive explanations (SHAP) values to visualize their contributions to the model predictions. Boruta is a random forest based algorithm for all relevant feature selection. It compares the importance of original genes with randomly generated shadow features. Genes that are classified as “selected” are retained (Kursa and Rudnicki, 2010). Finally, genes selected by all four algorithms were defined as the core genes.

### 2.7 Single-cell RNA sequencing analysis

To determine the cellular origin of the *PLAUR* signal identified by transcriptomic and machine learning analyses, and to characterize its distribution across tumor epithelial, immune, and stromal cells, we downloaded the breast cancer single-cell RNA sequencing dataset GSE161529 from the GEO database (Pal et al., 2021). Data processing was performed using the Seurat package in R (Stuart et al., 2019). When constructing the Seurat object, we retained genes expressed in at least three cells and cells with at least 200 detected genes. Cells with fewer than 200 detected genes were excluded because sparse expression profiles may represent cell debris, empty droplets, or low-quality cells. The data then underwent normalization, highly variable gene selection, principal component analysis, unsupervised clustering, and uniform manifold approximation and projection (UMAP) for dimensionality reduction.

Cell types were annotated using SingleR based on reference transcriptomic profiles. A density-based UMAP plot was generated to show the distribution density of annotated cell populations in the UMAP space, following a previously reported approach (Qiu et al., 2026). Finally, *PLAUR* expression was mapped onto each cell cluster, and its average expression level was calculated across clusters to evaluate its cell type-specific distribution.

### 2.8 Virtual overexpression of *PLAUR* and functional enrichment analysis

We simulated *PLAUR* overexpression in disease-derived single cells using the scTenifoldNet framework (Osorio et al., 2020). Subsampled gene regulatory networks were integrated through tensor decomposition. To create the perturbed network, we doubled the positive regulatory weights linked to *PLAUR*. The original and perturbed networks were compared by manifold alignment (Osorio et al., 2022). P values from the differential regulation analysis were adjusted using the BH method to control the FDR. Genes with an adjusted P < 0.05 were considered significantly perturbed. These genes were then analyzed with the clusterProfiler package for GO enrichment.

### 2.9 Molecular docking and molecular dynamics simulation

The 3D structure of BP-1 was obtained from the PubChem database. The protein structure of urokinase-type plasminogen activator receptor (uPAR), encoded by *PLAUR,* was downloaded from the RCSB Protein Data Bank (https://www.rcsb.org/). Molecular docking was performed using AutoDock Vina. The docking pose with the lowest binding energy was selected for visualization in PyMOL (https://www.pymol.org/). The selected docking pose was further analyzed using Discovery Studio Visualizer version 4.5 to characterize the predicted interactions between BP-1 and uPAR and generate the two-dimensional interaction diagram.

To evaluate the dynamic stability of the BP-1–uPAR complex, a 100 ns molecular dynamics simulation was carried out using GROMACS (version 2023.3). uPAR was parameterized with the AMBER99SB-ILDN force field. The complex was solvated in a periodic water box, neutralized with counterions, and energy-minimized. The system was first equilibrated under the constant-number, constant-volume, and constant-temperature (NVT) ensemble, followed by the constant-number, constant-pressure, and constant-temperature (NPT) ensemble, before production simulation. Visualizations were performed using DuIvyTools (version 0.6.0), and the following metrics were calculated: number of hydrogen bonds, root-mean-square deviation (RMSD), root-mean-square fluctuation (RMSF), radius of gyration (Rg), solvent-accessible surface area (SASA), and free energy landscape (He et al., 2025).

### 2.10 Analysis of *PLAUR* expression across cancers and in breast cancer

The pan-cancer expression profile of *PLAUR* was examined using the TIMER2.0 database (https://compbio.cn/timer2/). *PLAUR* expression was compared between tumor tissues and the corresponding normal tissues across multiple cancer types.

Transcriptomic data from The Cancer Genome Atlas breast cancer cohort (TCGA-BRCA) were obtained from the Genomic Data Commons portal (https://portal.gdc.cancer.gov/). *PLAUR* expression was compared between breast cancer tissues and normal tissues and visualized using a violin plot. Receiver operating characteristic (ROC) curve analysis was performed using the pROC package in R. The ability of *PLAUR* expression to distinguish breast cancer tissues from normal tissues was evaluated by calculating the area under the curve (AUC) and its 95% confidence interval.

### 2.11 Gene set enrichment analysis of *PLAUR*

TCGA-BRCA tumor samples were divided into high and low *PLAUR* expression groups using the median expression level. All genes were ranked according to the expression difference between the two groups. Gene set enrichment analysis (GSEA) was performed with the clusterProfiler package using GO and KEGG gene sets (Subramanian et al., 2005; Yu et al., 2012). The top five GO terms and KEGG pathways enriched in the high-*PLAUR* group were visualized using ridge plots.

### 2.12 Screening and functional enrichment analysis of *PLAUR*-correlated genes

To explore the potential functions of *PLAUR* in breast cancer, Spearman correlation analysis was performed to identify genes significantly correlated with *PLAUR* expression in the TCGA-BRCA dataset. Correlation coefficients (r) were calculated, and genes with |r| > 0.6 and P < 1 × 10^−5^ were selected for further analysis. Using the clusterProfiler package, GO and KEGG enrichment analyses were conducted on the correlated gene set, with significant terms filtered by adjusted P < 0.05 (BH correction). GO and KEGG results were visualized using circular and bubble plots, respectively.

### 2.13 Cell culture, macrophage differentiation, and treatment

MCF-7 and T-47D human breast cancer cells and THP-1 human monocytic cells were obtained from Procell Life Science & Technology Co., Ltd. (Wuhan, China). MCF-7 cells were cultured in DMEM (Gibco, Grand Island, NY, United States), whereas THP-1 and T-47D cells were cultured in RPMI-1640 medium (Gibco, Grand Island, NY, United States). All media were supplemented with 10% fetal bovine serum (FBS; CellMax, Lanzhou, China) and 1% penicillin-streptomycin (Gibco, Grand Island, NY, United States). Cells were maintained at 37 °C with 5% CO_2_.

THP-1 cells were treated with 100 ng/mL phorbol 12-myristate 13-acetate (PMA) for 24 h and then cultured in fresh complete medium for 24 h to obtain M0 macrophages (Lin et al., 2020). BP-1 was dissolved in DMSO, with a final DMSO concentration of 0.01% (v/v). M0 macrophages were treated with vehicle or BP-1 (1 or 10 μM) for 48 h.

For macrophage-conditioned medium (MCM) preparation, the treatment medium was removed after macrophage exposure, and the cells were washed three times with phosphate-buffered saline (PBS). Fresh complete RPMI-1640 medium without BP-1 was then added. After 24 h, the supernatants were collected as MCM.

For MCF-7 and T-47D experiments, MCM from macrophages exposed to vehicle was designated Vehicle-MCM and used as the control. MCM from macrophages exposed to 10 μM BP-1 was designated BP-1 (10 μM)-MCM. Vehicle-MCM supplemented with 10 μM BP-1 before breast cancer cell treatment was designated Vehicle-MCM + BP-1 (10 μM) and used as the direct BP-1 exposure control.

### 2.14 Cell viability assay

For the cell viability assay, THP-1 monocytes were seeded directly into 96-well plates before PMA differentiation (Browne et al., 2022). After differentiation and recovery as described in Section 2.13, M0 macrophages were treated with the indicated concentrations of BP-1 or vehicle for 48 h. Cell viability was measured using a Cell Counting Kit-8 (CCK-8; MedChemExpress, Monmouth Junction, NJ, United States) according to the manufacturer’s instructions. Absorbance was measured at 450 nm, and the half-maximal inhibitory concentration (IC_50_) was calculated from the dose-response curve.

For MCF-7 and T-47D cell viability assays, cells were seeded in 96-well plates and treated with BP-1 at 1 or 10 μM. Vehicle control wells received 0.01% DMSO. Cell viability was assessed at 0, 12, 24, 36, and 48 h using the same CCK-8 kit, and absorbance was measured at 450 nm.

### 2.15 Immunofluorescence staining

Macrophages treated as described in Section 2.13 were fixed, permeabilized, and blocked. The cells were then incubated overnight at 4 °C with primary antibodies against CD86 and CD206 (Proteintech, Wuhan, China), followed by fluorescent secondary antibodies (Thermo Fisher Scientific, Waltham, MA, United States). Nuclei were counterstained with DAPI (Beyotime, Shanghai, China), and images were captured on a confocal microscope (Carl Zeiss, Oberkochen, Germany).

### 2.16 Enzyme-linked immunosorbent assay

Culture supernatants were collected after 48 h of treatment. The concentrations of interleukin-10 (IL-10) and transforming growth factor beta 1 (TGF-β1) were measured using enzyme-linked immunosorbent assay (ELISA) kits (Multi Sciences, Hangzhou, China) according to the manufacturer’s instructions and calculated from standard curves.

### 2.17 Reverse transcription-quantitative PCR

Total RNA was extracted from macrophages and reverse transcribed into cDNA. Reverse transcription and quantitative PCR were performed using kits from Vazyme (Nanjing, China) with SYBR Green chemistry. The relative expression levels of *CD86*, *TNF*, *CD163*, *CD206*, and *TGFB1* were calculated using the 2^−ΔΔCT^ method, with β-actin (*ACTB*) as the reference gene.

### 2.18 EdU incorporation and colony formation assays

MCF-7 and T-47D cells were treated as described in Section 2.13. For the EdU incorporation assay, EdU was added to the culture medium at a final concentration of 10 μM, and cells were incubated for 2 h at 37 °C. The medium was then removed, and cells were fixed with 4% paraformaldehyde for 15 min at room temperature. After three washes, cells were permeabilized with 0.3% Triton X-100 in PBS for 15 min at room temperature. Following washing, cells were incubated with freshly prepared Click reaction mixture from the BeyoClick™ EdU-594 Cell Proliferation Kit (Beyotime, Shanghai, China) for 30 min at room temperature in the dark. Nuclei were counterstained with DAPI, and images were acquired using a confocal microscope (Carl Zeiss, Oberkochen, Germany). The percentage of EdU-positive cells was calculated as the number of EdU-positive cells divided by the total number of DAPI-positive nuclei × 100%.

For colony formation, cells were seeded at low density and cultured under the indicated conditions until visible colonies formed. Colonies were fixed, stained with crystal violet, and counted.

### 2.19 Cell migration and invasion assays

For migration assays, MCF-7 cells were seeded into uncoated Transwell inserts (Corning Inc., Corning, NY, United States); for invasion assays, cells were seeded into inserts coated with Matrigel. After 48 h, cells that had migrated or invaded were fixed, stained with crystal violet, and photographed using a light microscope (Leica Microsystems, Wetzlar, Germany). Four randomly selected, non-overlapping fields were photographed for each insert at the same magnification. The numbers of crystal violet-stained cells on the lower surface of the membrane were counted using ImageJ software (version 1.54f). The mean cell count from the four fields was used as the value for each insert.

### 2.20 Wound healing assay

After MCF-7 cells reached confluence, a linear scratch was made in the monolayer using a sterile pipette tip. Images were acquired at 0 and 24 h, and cell migration was evaluated by measuring the reduction in wound width.

### 2.21 Western blotting

Total protein was extracted from treated MCF-7 cells and macrophages and separated by SDS-PAGE. Proteins were transferred to membranes, and the membranes were blocked and then incubated overnight at 4 °C with primary antibodies. For MCF-7 samples, antibodies against E-cadherin, N-cadherin, vimentin, Snail, and GAPDH were used; for macrophage samples, antibodies against uPAR and GAPDH were used (all from Proteintech, Wuhan, China). The membranes were then incubated with HRP-conjugated secondary antibodies (Proteintech). Blots were developed using an enhanced chemiluminescence substrate (NCM Biotech, Suzhou, China), and band intensities were quantified using ImageJ, with GAPDH as the loading control.

### 2.22 Statistical analysis

Statistical analyses were performed using GraphPad Prism (version 10.0.0). Comparisons between two groups were conducted using Student’s t-test, while comparisons among three groups were assessed via one-way ANOVA followed by the Tukey–Kramer *post hoc* test. All experiments were carried out in triplicate, and data are expressed as mean ± standard deviation (SD). *P* < 0.05 was considered statistically significant.

## 3 Results

### 3.1 Integrated transcriptomic and WGCNA analyses identify breast cancer-related genes

To improve comparability across GSE42568, GSE45827, and GSE70905, batch correction was performed before analysis. Batch correction aligned the overall expression distributions of the three datasets and reduced their separation in the PCA space (Figures 1A–D). Using |log_2_FC| > 0.585 and adjusted P < 0.05 as cutoff criteria, we screened 1,549 DEGs between breast cancer and normal samples (Figures 1E,F). We then performed WGCNA with a soft-thresholding power of three and identified 13 co-expression modules (Figures 1G,H). Among these, the co-expression module showing the strongest association with breast cancer (P = 2 × 10^−28^) was selected for further analysis (Figure 1I). This module contained 85 genes. The union of the DEG set and these 85 genes yielded a final set of 1,597 breast cancer-related genes for subsequent analyses (Figure 1J).

**FIGURE 1:**
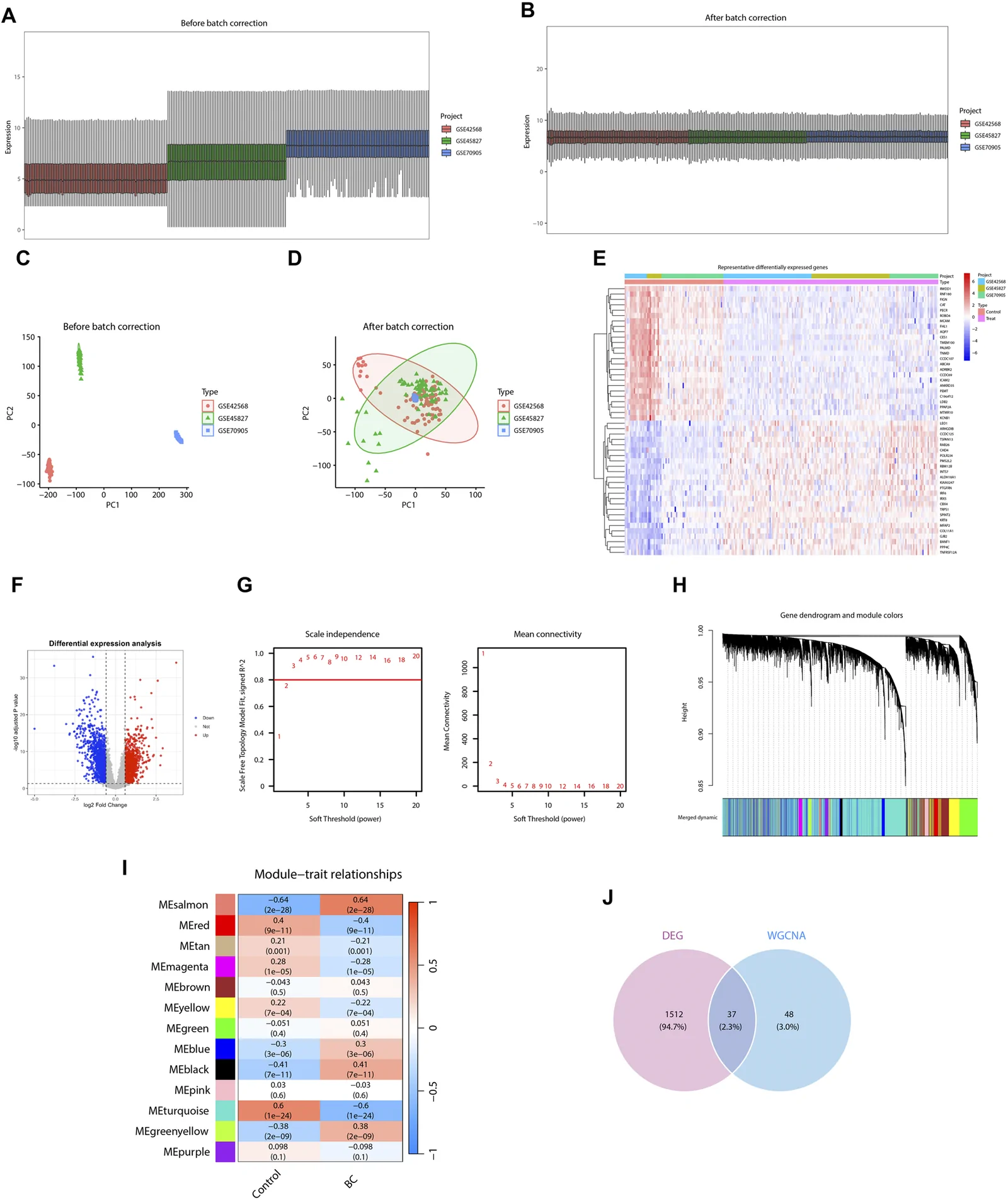
Identification of breast cancer-related genes by integrated transcriptomic analysis and WGCNA. **(A,B)** Expression distributions of samples from GSE42568, GSE45827, and GSE70905 before and after batch correction. **(C,D)** PCA plots before and after batch correction. **(E)** Heatmap of representative DEGs between breast cancer and normal samples. **(F)** Volcano plot of DEGs identified using |log_2_FC| > 0.585 and adjusted P < 0.05. **(G)** Selection of the soft-thresholding power based on scale-free topology fit and mean connectivity. **(H)** Gene clustering dendrogram and corresponding module assignments. **(I)** Correlations between module eigengenes and breast cancer status. The co-expression module showing the strongest association with breast cancer was selected for subsequent analysis. **(J)** Overlap between 1,549 DEGs and 85 genes from the selected co-expression module. The union of the two gene sets yielded 1,597 breast cancer-related genes. DEG, differentially expressed gene; PCA, principal component analysis; WGCNA, weighted gene co-expression network analysis.

### 3.2 Physicochemical properties and target identification of BP-1 in breast cancer

The 2D and 3D structures of BP-1 are displayed in Figures 2A,B. The molecular properties of BP-1 derived from ProTox 3.0 are summarized in Figure 2C. Toxicity target prediction pointed to possible interactions with estrogen receptor α and its ligand binding domain, mitochondrial membrane potential, transthyretin, and several cytochrome P450 enzymes (Figure 2D).

**FIGURE 2:**
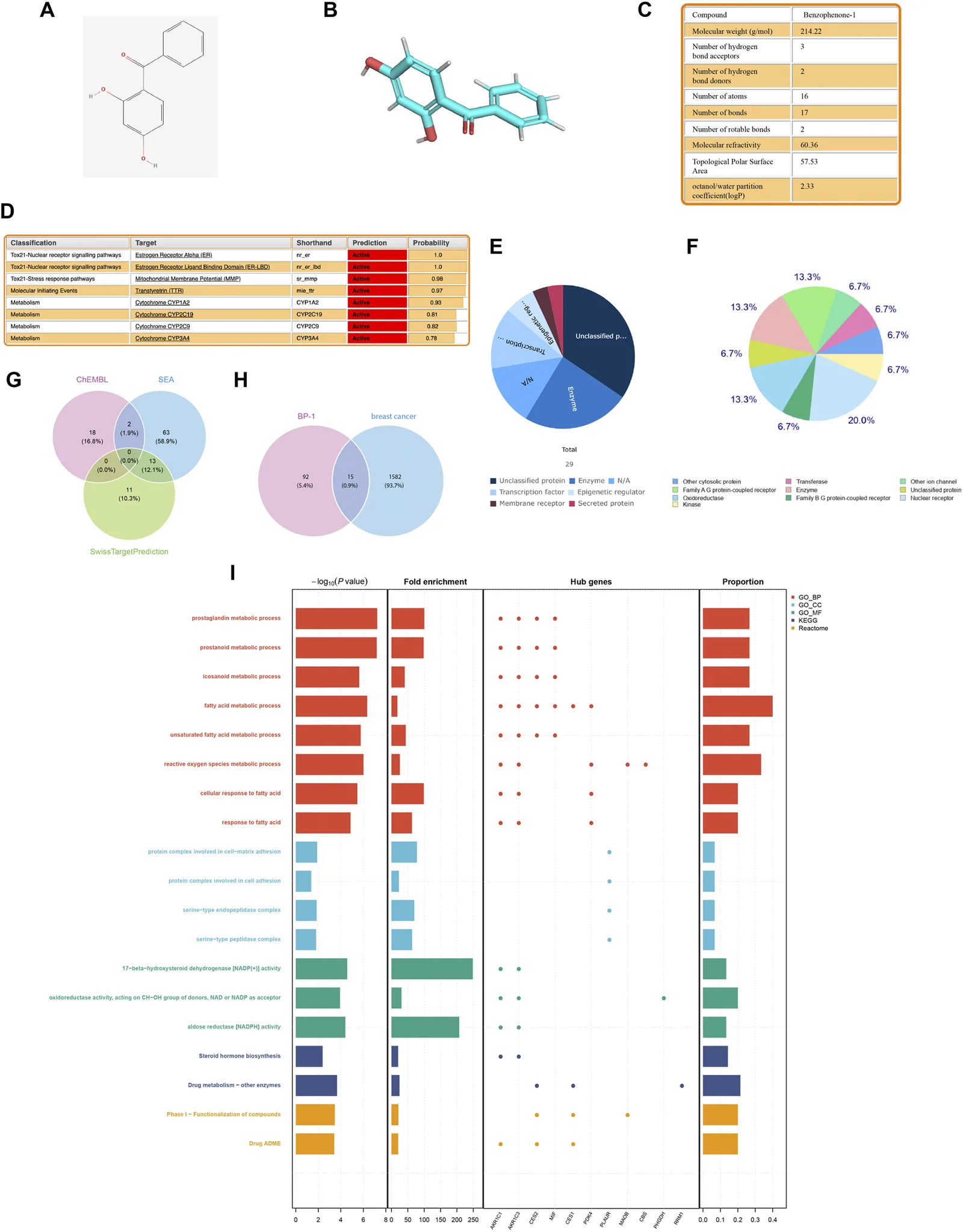
Chemical characterization, toxicological target prediction, and target identification of BP-1 in breast cancer. **(A,B)** 2D and 3D structures of BP-1 (Panel 2A adapted from National Center for Biotechnology Information). **(C)** Molecular properties of BP-1. **(D)** Predicted toxicological and metabolic activities of BP-1 (Panel 2C,D adapted from Banerjee et al. (2024)). **(E–G)** Target prediction of BP-1 using ChEMBL, SEA, and SwissTargetPrediction (Panel 2E adapted from Zdrazil et al. (2023); (Panel 2F adapted from Keiser et al. (2007)). **(H)** Venn diagram showing 15 shared candidate genes between 107 BP-1-related targets and 1,597 breast cancer-related genes. **(I)** GO, KEGG, and Reactome enrichment analysis of the 15 candidate genes. BP-1, benzophenone-1; GO, Gene Ontology; KEGG, Kyoto Encyclopedia of Genes and Genomes; SEA, Similarity Ensemble Approach.

We then integrated data from ChEMBL, SEA, and SwissTargetPrediction, which yielded 107 BP-1-associated targets (Figures 2E–G). Fifteen of these overlapped with the 1,597 breast cancer-related genes we had previously identified, and these genes were retained as candidate genes (Figure 2H). Functional enrichment analysis further revealed that these candidates were mainly enriched in prostaglandin, fatty acid, and reactive oxygen species metabolism, cell adhesion-related protein complexes, steroid hormone biosynthesis, and drug metabolism (Figure 2I).

### 3.3 Machine learning prioritizes *PLAUR* as the core gene

To evaluate the 15 candidate genes, four machine learning algorithms were applied. LASSO kept genes with nonzero coefficients at the optimal penalty parameter (Figures 3A,B). SVM-RFE selected seven features, with a minimum cross-validation error of 0.147 and a maximum accuracy of 0.853 (Figures 3C,D). XGBoost ranked *CA3*, *PLAUR*, *AKR1C1*, *ABAT*, and *CES1* as the top five features, and we used SHAP analysis to further examine their contributions (Figures 3E,F). Boruta analysis confirmed the relevance of the candidate set (Figures 3G,H). Consistently prioritized across the four algorithms, *PLAUR* emerged as the core gene and was selected for subsequent analyses (Figure 3I).

**FIGURE 3:**
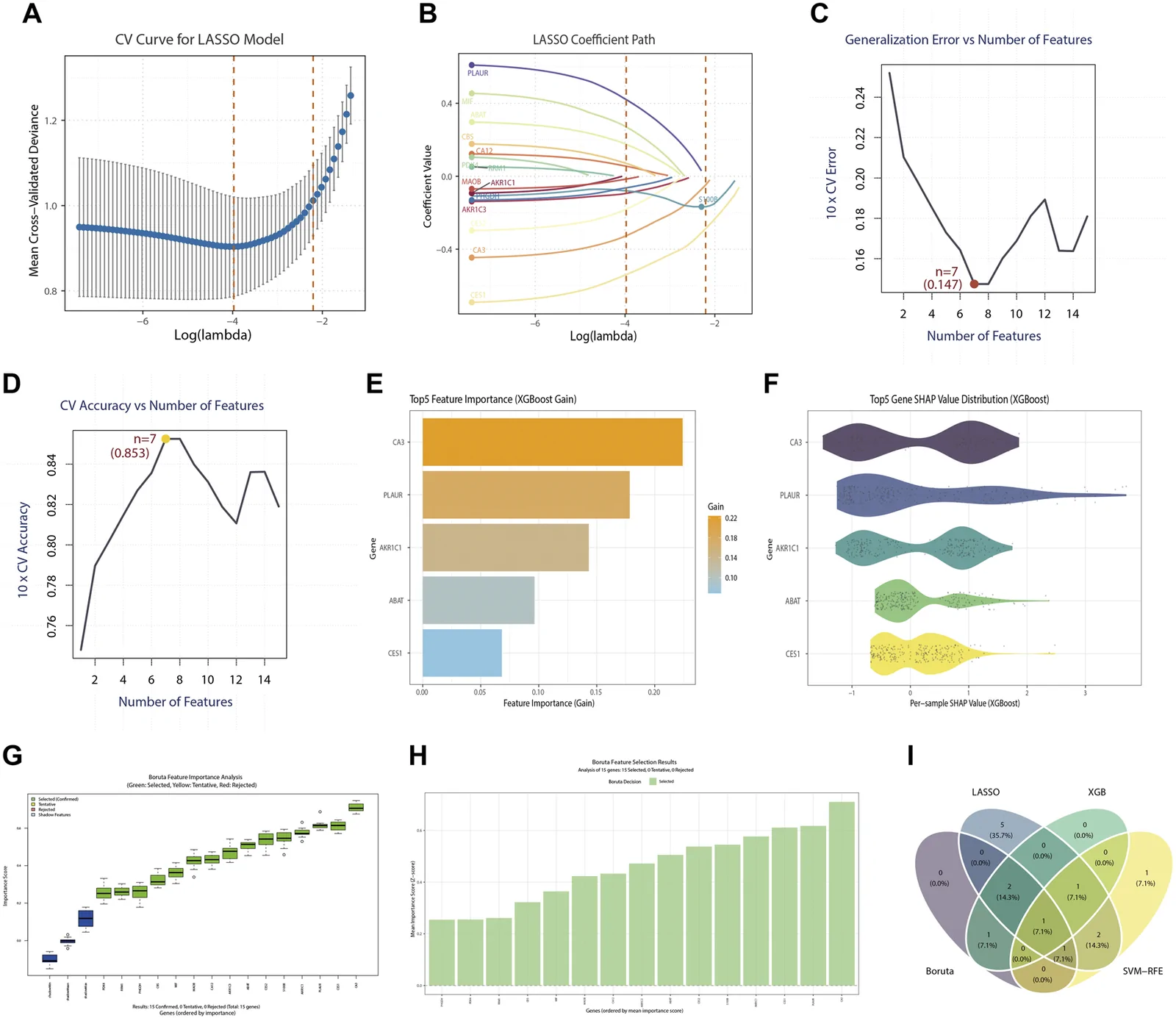
Machine learning prioritizes PLAUR as the core gene. **(A)** Cross-validation curve of the LASSO model. **(B)** LASSO coefficient profiles of the 15 candidate genes. **(C,D)** Cross-validation error and accuracy of the SVM-RFE model according to the number of selected features. The optimal model included seven features. **(E)** Top five features ranked by XGBoost gain. **(F)** SHAP value distributions of the top five XGBoost features. **(G,H)** Feature importance and selection results from Boruta analysis. **(I)** Overlap of the genes selected by LASSO, SVM-RFE, XGBoost, and Boruta, prioritizing PLAUR as the core gene. LASSO, least absolute shrinkage and selection operator; SHAP, Shapley additive explanations; SVM-RFE, support vector machine recursive feature elimination; XGBoost, extreme gradient boosting.

### 3.4 Single-cell distribution and virtual overexpression analysis of *PLAUR*

SingleR annotation of the GSE161529 single-cell dataset revealed seven major cell types, including epithelial cells, T cells, B cells, macrophages, fibroblasts, endothelial cells, and tissue stem cells (Figure 4A). A density-based Nebula plot was used to show the relative density and distribution of the annotated cell populations in UMAP space (Figure 4B). *PLAUR* was mainly expressed in macrophage, epithelial cell, and fibroblast clusters (Figure 4C). Virtual overexpression of *PLAUR* led to substantial changes in *VIM*, *OLFML3*, *TIMP2*, *HSP90AA1*, *HTRA1*, *LRP1*, and *MARCKS*, with *VIM* showing the strongest statistical signal (Figure 4D). These genes were mainly linked to peptidase regulation, focal adhesion, cell–substrate junctions, collagen-containing extracellular matrix, and intermediate filament binding (Figure 4E).

**FIGURE 4:**
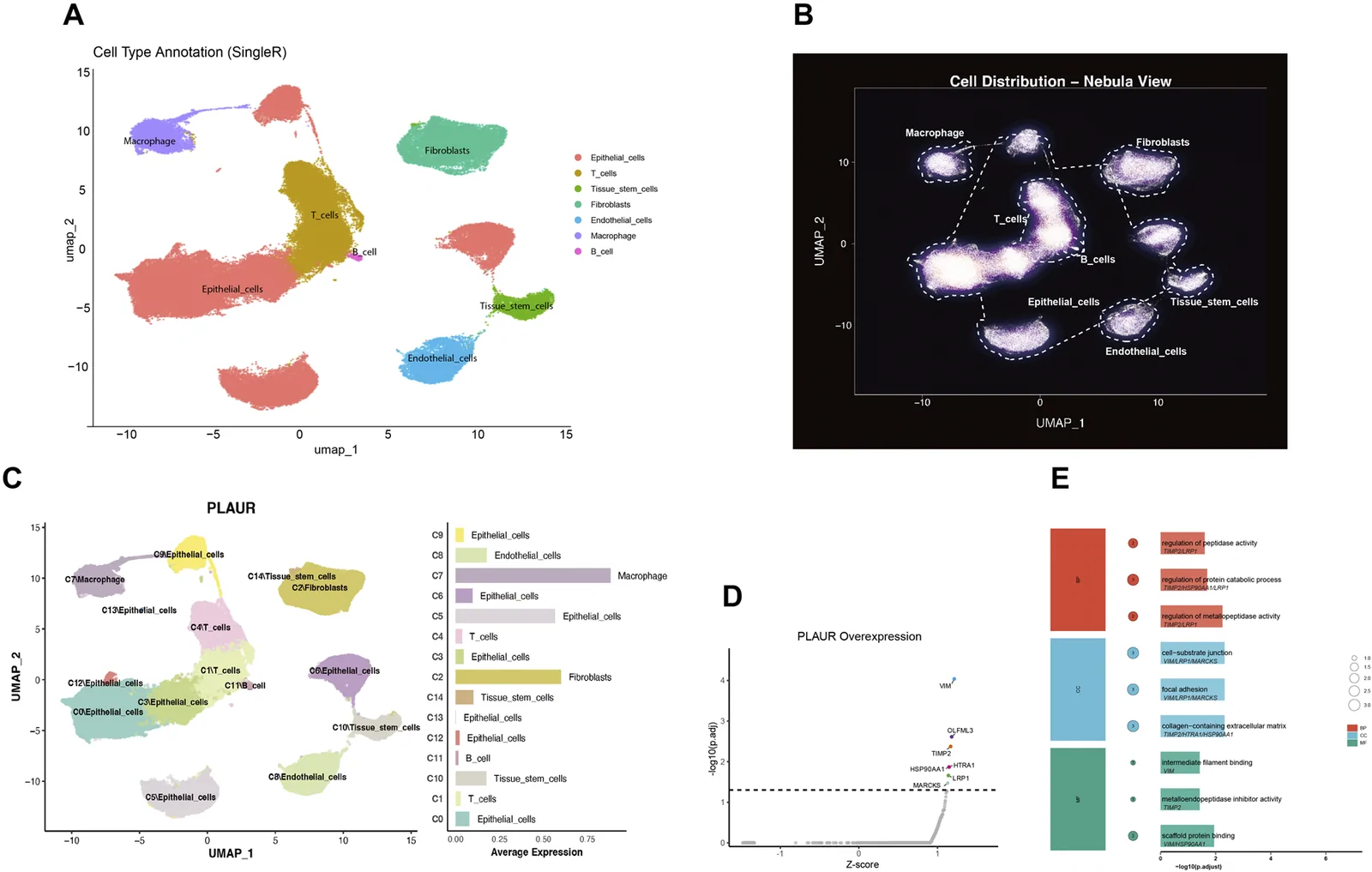
Single-cell distribution and virtual overexpression analysis of *PLAUR*. **(A)** UMAP plot of GSE161529 cells annotated by SingleR. **(B)** Density-based visualization of the annotated cell populations. **(C)** Distribution and average expression of PLAUR across cell clusters. PLAUR showed the highest average expression in the macrophage cluster. **(D)** Genes significantly perturbed following virtual PLAUR overexpression. The dashed line indicates adjusted *P* = 0.05. **(E)** GO enrichment analysis of genes significantly perturbed by virtual PLAUR overexpression. BP, biological process; CC, cellular component; GO, Gene Ontology; MF, molecular function; UMAP, uniform manifold approximation and projection.

### 3.5 Molecular docking and dynamics of the BP-1–uPAR complex

Molecular docking revealed that BP-1 bound to uPAR, with a binding energy of −6.9 kcal/mol (Figure 5A). The two-dimensional interaction diagram illustrates the predicted interactions between BP-1 and multiple residues within the uPAR binding pocket, including hydrogen bonds, π interactions, and van der Waals contacts (Figure 5B). To evaluate the dynamic stability of this complex, a 100 ns molecular dynamics simulation was performed. After the initial equilibration, the RMSD quickly plateaued and remained mostly within 0.6–0.9 nm (Figure 5C). RMSF analysis indicated low flexibility, with most atomic fluctuations below 0.6 nm, suggesting a stable local structure (Figure 5D). The Rg stayed nearly constant at approximately 2.2–2.4 nm, indicating that the overall compactness was preserved over the simulation (Figure 5E). The SASA fluctuated mainly between 145 and 160 nm^2^, showing no notable change in solvent exposure (Figure 5F). Hydrogen bond analysis revealed that the complex typically formed 1–2 hydrogen bonds, with a maximum of 4 (Figure 5G). Finally, the free energy landscape displayed a prominent low-energy basin (Figures 5H,I), further supporting the conformational stability of the BP-1–uPAR complex.

**FIGURE 5:**
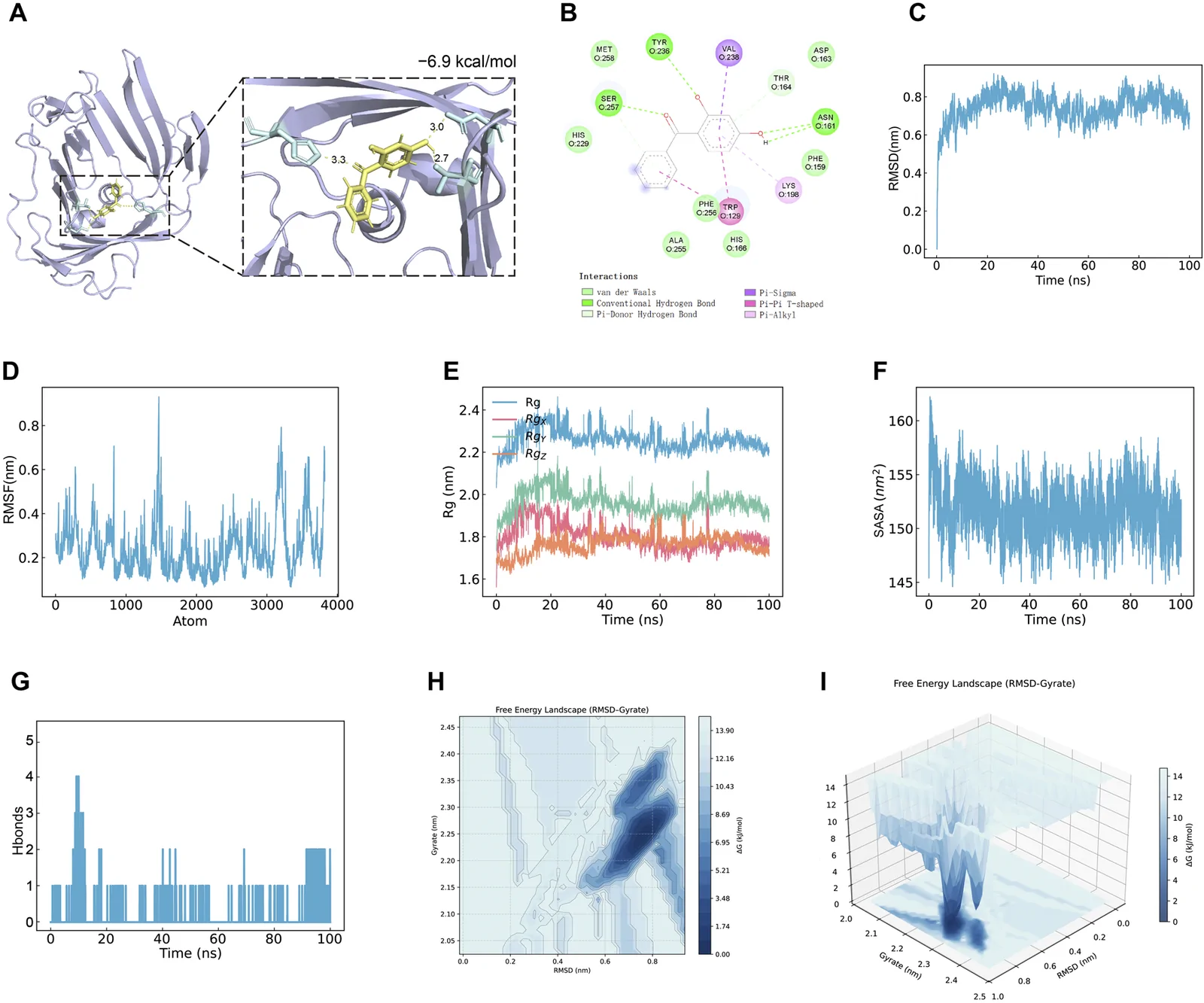
Molecular docking and molecular dynamics analysis of the BP-1–uPAR complex. **(A)** Molecular docking predicted binding of BP-1 to uPAR, with a binding energy of −6.9 kcal/mol. **(B)** Two-dimensional interaction diagram showing the predicted interactions between BP-1 and residues in the uPAR binding pocket. **(C)** RMSD during the 100 ns simulation. **(D)** RMSF plotted against atom number. **(E)** Rg and its components along the x, y, and z axes. **(F)** SASA. **(G)** Number of hydrogen bonds formed between BP-1 and uPAR during the simulation. **(H,I)** Two-dimensional **(H)** and three-dimensional **(I)** free energy landscapes based on RMSD and Rg. BP-1, benzophenone-1; uPAR, urokinase-type plasminogen activator receptor; RMSD, root-mean-square deviation; RMSF, root-mean-square fluctuation; Rg, radius of gyration; SASA, solvent-accessible surface area.

### 3.6 *PLAUR* is upregulated and associated with immune responses and extracellular matrix remodeling in breast cancer

Pan-cancer analysis revealed significant differences in *PLAUR* expression between tumor and normal tissues across multiple cancer types (Figure 6A). In the TCGA-BRCA cohort, *PLAUR* expression was higher in tumor tissues than in normal tissues (*P* = 1.494 × 10^−41^), with an AUC of 0.885 from ROC analysis (Figures 6B,C). GSEA revealed that the high-*PLAUR* group was mainly enriched in gene sets involved in immune and inflammatory signaling, antigen processing and presentation, and cell migration (all FDR < 0.001; Figures 6D,E). Spearman correlation analysis of the TCGA-BRCA dataset further demonstrated strong positive correlations between *PLAUR* and *PLAU*, *TGFBI*, and *SPHK1*, with correlation coefficients of 0.71, 0.69, and 0.66, respectively (all *P* < 0.001; Figures 6F–H). GO and KEGG enrichment analyses linked the *PLAUR*-correlated gene set to cell adhesion, extracellular matrix remodeling, mesenchymal differentiation, proteolysis, and immune-related processes (Figures 6I,J).

**FIGURE 6:**
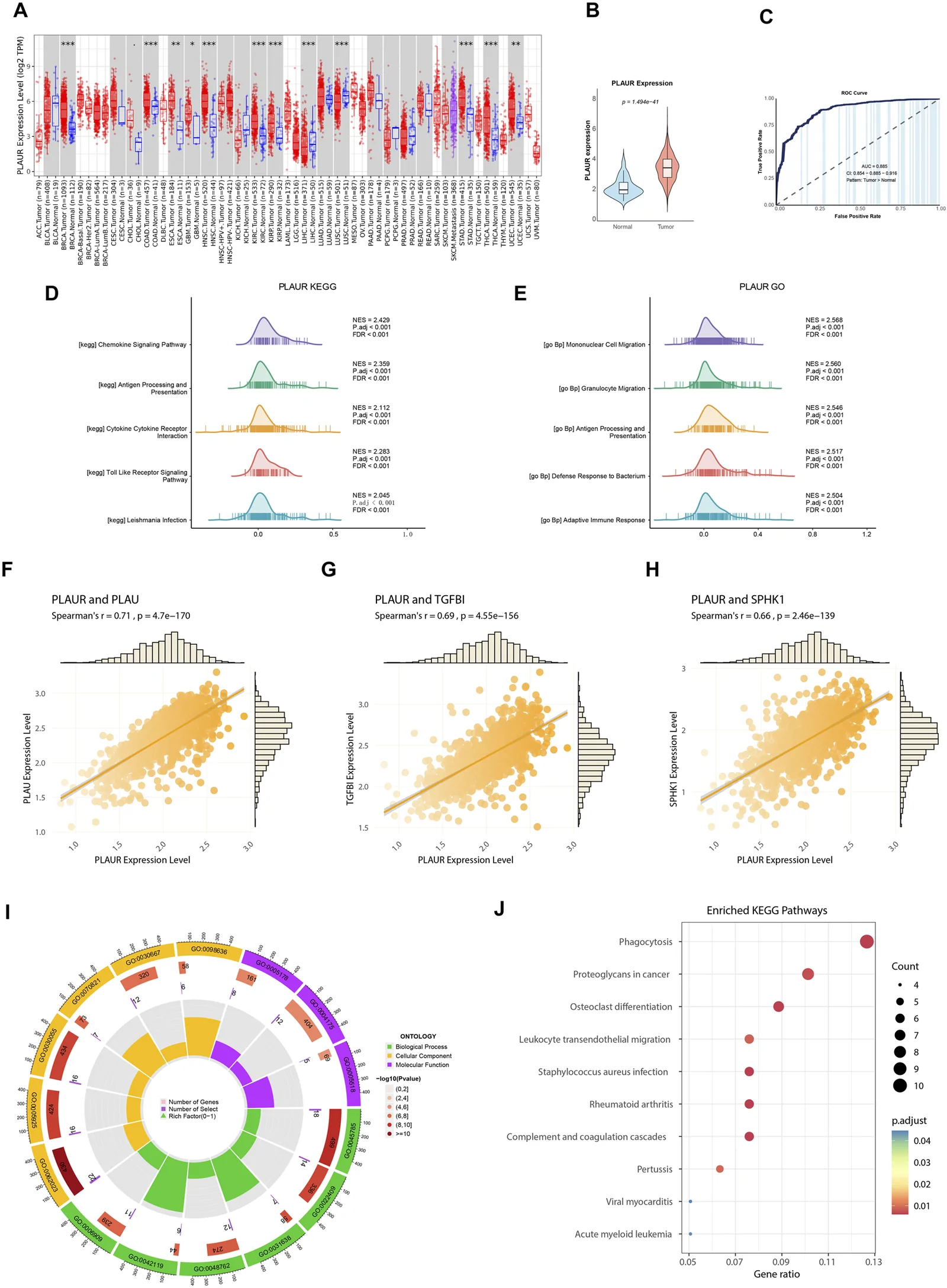
Expression and functional associations of *PLAUR* in breast cancer. **(A)** Pan-cancer comparison of PLAUR expression between tumor and normal tissues using TIMER2.0. **(B)** PLAUR expression levels in TCGA-BRCA tumor and normal tissues. **(C)** ROC curve evaluating the ability of *PLAUR* expression to distinguish breast cancer from normal tissue (AUC = 0.885). **(D,E)** GSEA enrichment analysis showing KEGG pathways **(D)** and GO biological processes **(E)** enriched in the high-PLAUR group. **(F–H)** Spearman correlations of PLAUR with *PLAU* **(F)**, *TGFBI* **(G)**, and *SPHK1* **(H)**. **(I,J)** GO functional enrichment and KEGG pathway enrichment of PLAUR-correlated genes. *P < 0.05, **P < 0.01, ***P < 0.001. TIMER2.0: Tumor Immune Estimation Resource 2.0; TCGA-BRCA: The Cancer Genome Atlas Breast Invasive Carcinoma; ROC: receiver operating characteristic; AUC, area under the curve; GSEA, gene set enrichment analysis; GO, Gene Ontology; KEGG, Kyoto Encyclopedia of Genes and Genomes.

### 3.7 BP-1 exposure shifts THP-1-derived macrophages toward an M2-like phenotype

*PLAUR* showed prominent expression in macrophage clusters and was associated with immune and inflammatory processes. We therefore examined whether BP-1 alters the polarization of THP-1-derived macrophages, a functional change involved in tumor microenvironment remodeling. After 48 h of exposure, the IC_50_ of BP-1 was 118.5 μM. Previous *in vitro* studies of BP-1 have included exposure concentrations of 1 and 10 μM (In et al., 2015). In the present study, 1 and 10 μM BP-1 did not significantly affect the viability of THP-1-derived macrophages after 48 h and were well below the IC_50_. These concentrations were therefore used in subsequent assays (Figure 7A). Western blotting showed that uPAR protein expression increased with BP-1 concentration (Figures 7B,C). TGF-β1 and IL-10 secretion also increased in a concentration-dependent manner (Figures 7D,E). Immunofluorescence staining showed decreased CD86 and increased CD206 signals with increasing BP-1 concentrations (Figure 7F). At the mRNA level, *CD86* decreased at both concentrations, whereas *TNF* was significantly reduced only at 10 μM. In contrast, *CD163*, *CD206*, and *TGFB1* increased with BP-1 concentration (Figures 7G–K). These changes in phenotype and secretion suggest that BP-1 skewed THP-1-derived macrophages toward an M2-like phenotype.

**FIGURE 7:**
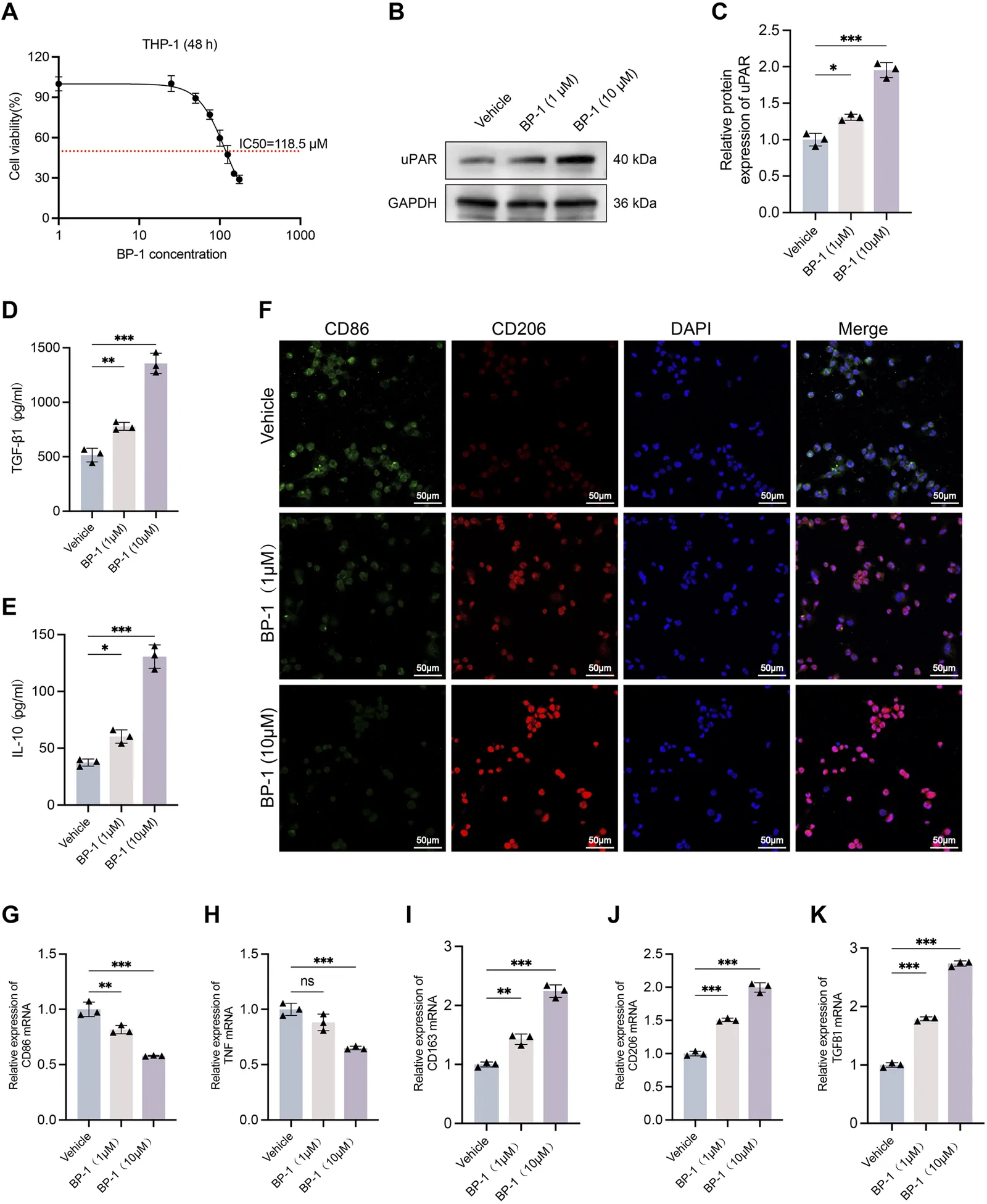
BP-1 exposure shifts THP-1-derived macrophages toward an M2-like phenotype. **(A)** CCK-8 dose–response curve of THP-1-derived macrophages exposed to BP-1 for 48 h. The calculated IC_50_ was 118.5 μM. **(B,C)** Representative western blot bands **(B)** and densitometric quantification of uPAR normalized to GAPDH **(C)** in macrophages treated with vehicle or BP-1 (1 or 10 μM) for 48 h. **(D,E)** Concentrations of TGF-β1 **(D)** and IL-10 **(E)** in culture supernatants determined by ELISA. **(F)** Representative immunofluorescence images of CD86 (green) and CD206 (red). Nuclei were counterstained with DAPI (blue). **(G–K)** Relative mRNA expression of *CD86* **(G)**, *TNF* **(H)**, *CD163* **(I)**, *CD206* **(J)**, and *TGFB1* **(K)**. Data are presented as the mean ± SD (n = 3). ns, not significant; *P < 0.05, **P < 0.01, and ***P < 0.001. BP-1, benzophenone-1; CCK-8, Cell Counting Kit-8; IC_50_, half-maximal inhibitory concentration; DAPI, 4′,6-diamidino-2-phenylindole; ELISA, enzyme-linked immunosorbent assay.

### 3.8 BP-1-treated macrophage-conditioned medium enhances breast cancer cell proliferation and invasiveness

Having observed an M2-like shift and increased TGF-β1 secretion in BP-1-treated macrophages, we next examined whether their conditioned medium affected breast cancer cell behavior. MCF-7 and T-47D cells were used to evaluate whether the effects of BP-1-treated MCM were reproducible across two breast cancer cell lines. The CCK-8 assay showed that direct BP-1 treatment at 1 and 10 μM did not reduce the viability of MCF-7 or T-47D cells (Supplementary Figure S1).

In MCF-7 cells, MCM from macrophages treated with 10 μM BP-1 significantly increased the proportion of EdU-positive cells, indicating that more cells were undergoing DNA synthesis (Figures 8A,B). A comparable increase was observed in T-47D cells, showing that this proliferative response was not restricted to MCF-7 cells (Supplementary Figures S2A,B). To determine whether increased DNA synthesis was accompanied by sustained proliferative capacity, colony formation assays were performed. BP-1-treated MCM significantly increased colony formation in both MCF-7 and T-47D cells, indicating that more cells retained the ability to survive and undergo successive divisions over the assay period (Figures 8C,D; Supplementary Figures S2C,D).

**FIGURE 8:**
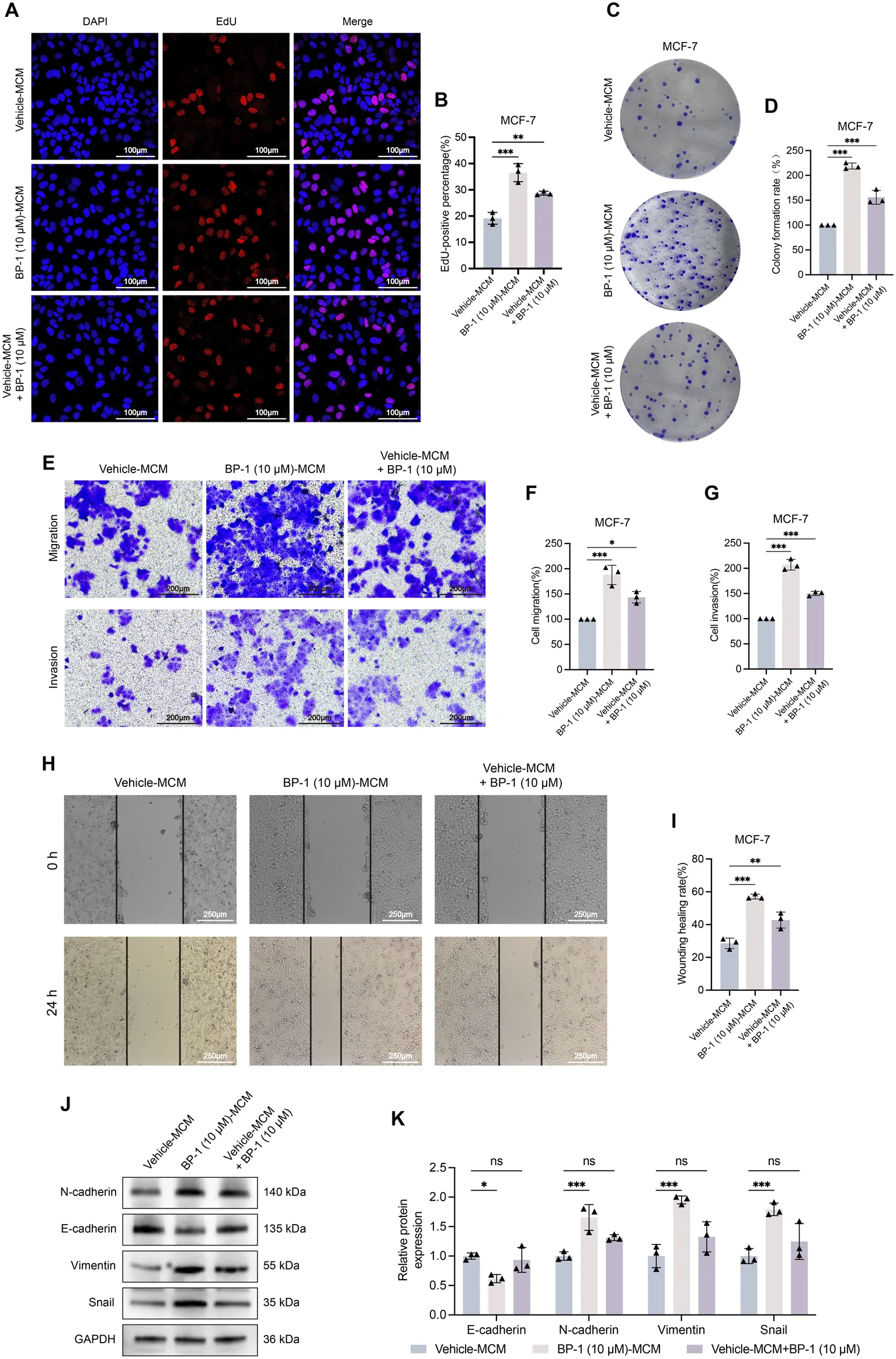
BP-1-treated macrophage-conditioned medium enhances proliferation and invasiveness in MCF-7 cells. **(A,B)** Representative EdU staining images **(A)** and percentage of EdU-positive cells **(B)**. EdU-positive cells are shown in red, and nuclei were counterstained with DAPI (blue). **(C,D)** Representative colony formation images **(C)** and corresponding quantification **(D)**. **(E–G)** Representative transwell migration and invasion images **(E)** and quantification of migrated **(F)** and invaded **(G)** cells. **(H,I)** Representative wound healing images at 0 and 24 h **(H)** and corresponding quantification **(I)**. **(J,K)** Western blot analysis of E-cadherin, N-cadherin, Vimentin, and Snail **(J)** and densitometric quantification normalized to GAPDH **(K)**. Data are presented as the mean ± SD (n = 3). ns, not significant; *P < 0.05, **P < 0.01, and ***P < 0.001. BP-1, benzophenone-1; MCM, macrophage-conditioned medium; EdU, 5-ethynyl-2′-deoxyuridine.

Transwell assays showed that BP-1-treated MCM increased the numbers of migrated and invaded MCF-7 cells, reflecting enhanced motility and an increased ability to traverse the extracellular matrix barrier (Figures 8E–G). Consistently, BP-1-treated MCM accelerated wound closure over 24 h in the wound healing assay (Figures 8H,I). Vehicle-MCM + BP-1 (10 μM) significantly increased EdU incorporation, colony formation, migration, invasion, and wound closure compared with Vehicle-MCM (all P < 0.05). By contrast, Vehicle-MCM + BP-1 (10 μM) did not significantly alter the expression of E-cadherin, N-cadherin, vimentin, or Snail compared with Vehicle-MCM (all P > 0.05).

Finally, western blotting was used to determine whether these functional changes were accompanied by an EMT-related molecular shift. BP-1-treated MCM decreased the epithelial marker E-cadherin and increased the mesenchymal markers N-cadherin and vimentin, together with the EMT-related transcription factor Snail (Figures 8J,K). Direct BP-1 exposure, however, did not significantly alter the expression of any of these proteins compared with vehicle-MCM.

Taken together, BP-1-treated MCM enhanced proliferative activity in two ER-positive breast cancer cell lines and promoted migration, invasion, and EMT-related protein changes in MCF-7 cells. Along with the M2-like polarization and increased TGF-β1 secretion observed in macrophages, these results support a macrophage-associated contribution to the effects of BP-1 on breast cancer cells.

## 4 Discussion

This study investigated whether BP-1 alters breast cancer cell behavior through changes in macrophage phenotype and secretion. BP-1 shifted THP-1-derived macrophages toward an M2-like phenotype, and conditioned medium from these cells enhanced breast cancer cell proliferation, migration, and invasion and altered EMT-related protein expression. These findings indicate that macrophage reprogramming contributes to BP-1-induced changes in these malignant phenotypes.

*PLAUR* was identified as the core BP-1-related candidate, and single-cell analysis showed prominent *PLAUR* expression in macrophage clusters, providing a rationale for examining its response in macrophages. Molecular docking and molecular dynamics simulations predicted a stable binding mode between BP-1 and uPAR, the protein encoded by *PLAUR*. In THP-1-derived macrophages, uPAR protein expression increased with BP-1 concentration, showing that the computationally prioritized target was also responsive to BP-1 at the protein level. These observations suggest that uPAR may participate in the macrophage response to BP-1.

BP-1 increased the mRNA expression of *CD163*, *CD206*, and *TGFB1*, while decreasing *CD86* and *TNF*. ELISA showed that BP-1-treated macrophages secreted more IL-10 and TGF-β1. These changes are consistent with a shift toward an M2-like phenotype. In tumors, M2-like macrophages generally exhibit anti-inflammatory and immunoregulatory functions. More broadly, they can establish a tumor-supportive microenvironment by promoting tumor cell growth and survival, tissue and extracellular matrix remodeling, invasion and metastasis, angiogenesis, and immune evasion (Mehta et al., 2021). Among the factors elevated after BP-1 treatment, TGF-β1 may provide a potential link between macrophage reprogramming and the EMT-related changes observed in breast cancer cells (Derynck et al., 2021). Upon ligand binding, the TGF-β receptor complex phosphorylates SMAD2/3, which then associates with SMAD4 and induces EMT-related transcription factors such as Snail. Snail, in turn, represses E-cadherin expression. TGF-β1 also activates non-SMAD pathways, including MAPK, PI3K/AKT, and Rho GTPases, which regulate cytoskeletal organization and cell motility (Derynck et al., 2021; Massagué, 2012). In breast cancer models, TGF-β1-induced EMT has been shown to enhance cell migration, while MCM can induce TGF-β-dependent EMT in MCF-7 cells (Pang et al., 2016; Kundu and Shankar, 2022). These findings are consistent with the reduced E-cadherin, increased Snail, N-cadherin, and vimentin, and enhanced migration and invasion observed in MCF-7 cells exposed to BP-1-treated MCM. By contrast, direct BP-1 exposure did not significantly alter these EMT-related proteins, supporting a contribution from the altered macrophage secretome.

Several of our findings are consistent with previous experimental studies. BP-1 has been reported to stimulate MCF-7 cell proliferation through ERα-dependent signaling (In et al., 2015), while exposure to 1 and 10 μM BP-1 increased both proliferation and colony formation, particularly after prolonged treatment (Confortin et al., 2026). The increased EdU incorporation and clonogenic growth in our direct-exposure group therefore agree with the reported proliferative effects of BP-1 in ER-positive breast cancer cells. A similar response was obtained in T-47D cells, indicating that this effect was not confined to MCF-7 cells. Long-term BP-1 exposure has also been reported to enhance MCF-7 cell motility and invasiveness (Alamer and Darbre, 2018). Although the exposure duration was substantially longer than that used here, the direction of the response agrees with the increases observed in our direct-exposure group. At the molecular level, *PLAUR* was identified as a core candidate associated with BP-1 and malignant phenotypes in breast cancer, particularly invasion and extracellular matrix remodeling. Previous studies have shown that signaling between uPAR and integrins promotes breast cancer cell invasion through the extracellular matrix, whereas uPAR depletion reduces this response (Annis et al., 2018). This agrees with the repeated enrichment of biological processes related to cell migration and extracellular matrix remodeling in our analyses. Importantly, the assays used here examined distinct aspects of cell behavior: EdU incorporation and colony formation assessed proliferative activity over different time scales. Increased migration was observed in both the Transwell and wound healing assays, while the Matrigel invasion assay further demonstrated an increased ability to cross an extracellular matrix barrier. The consistency across these complementary readouts and two breast cancer cell lines supports the robustness of the observed proliferative and motility phenotypes.

Earlier breast cancer studies mainly examined BP-1 through direct exposure of tumor cells (Kerdivel et al., 2013; In et al., 2015). By comparing direct BP-1 exposure with MCM from BP-1-treated macrophages, the present study identifies an additional macrophage-associated component. This extends the interpretation of BP-1 activity beyond its direct estrogenic effects on breast cancer cells.

A strength of this study was the direct comparison between BP-1-treated MCM and vehicle MCM supplemented with BP-1, which helped distinguish macrophage-associated responses from the effects of direct exposure. The agreement between computational target identification and the increase in uPAR protein expression provides a basis for subsequent mechanistic studies.

Some limitations should be considered. Firstly, the findings were obtained using THP-1-derived macrophages and predominantly MCF-7 cells, and validation in primary macrophages and additional breast cancer models would strengthen their generalizability. Secondly, uPAR and TGF-β1 emerged as potential mediators, but further experiments involving *PLAUR* perturbation and TGF-β1 neutralization are needed to clarify their roles. Thirdly, ER signaling was not examined using an ER antagonist or receptor knockdown. Its contribution to the responses to direct BP-1 and MCM could therefore not be determined.

These findings suggest that macrophage responses warrant further investigation when evaluating the biological effects of ultraviolet filters. Sunscreen use nevertheless remains important for preventing ultraviolet damage and skin cancer (Green et al., 2011). Concerns about conventional organic filters have increased interest in newer compounds such as bemotrizinol, a broad-spectrum triazine filter with greater photostability and low systemic exposure following topical application (D'Ruiz et al., 2023). However, improved photoprotection does not necessarily ensure biological or environmental safety. The effects of new filters, their metabolites, and repeated exposure should therefore be evaluated before they are widely used to replace existing benzophenone filters.

## 5 Conclusion

In conclusion, this study combined computational analyses with experimental validation to examine the effects of BP-1 in breast cancer. *PLAUR* was identified as a core candidate gene linked to BP-1 and showed high expression in macrophage clusters. Genes associated with *PLAUR* were enriched in immune and inflammatory processes. Molecular docking and molecular dynamics simulations suggested an interaction between BP-1 and uPAR. These analyses highlighted *PLAUR*/uPAR as a candidate molecular link in the actions of BP-1.

In macrophages differentiated from THP-1 cells, BP-1 exposure induced changes consistent with a shift toward an M2-like phenotype. It increased the expression of *CD163*, *CD206*, and *TGFB1* and reduced the expression of *CD86* and *TNF*. BP-1 exposure also increased the secretion of IL-10 and TGF-β1. MCM obtained from BP-1-exposed macrophages increased proliferative activity and promoted malignant phenotypes, including migration, invasion, wound closure, and EMT-related changes, in breast cancer cells. Overall, these findings suggest that BP-1 may promote breast cancer progression through macrophage reprogramming and changes in macrophage-secreted factors.

## Data Availability

The datasets presented in this study can be found in online repositories. The names of the repository/repositories and accession number(s) can be found in the article/Supplementary Material.

## Supplementary material

SUPPLEMENTARY FIGURE S1 Effects of BP-1 on the viability of MCF-7 and T-47D cells. **(A)** MCF-7 cells and **(B)** T-47D cells were treated with vehicle or BP-1 (1 or 10 μM) for 0, 12, 24, 36, and 48 h. Data are presented as the mean ± SD (n = 3). ns, not significant; *P < 0.05, **P < 0.01, and ***P < 0.001. BP-1: benzophenone-1.

SUPPLEMENTARY FIGURE S2 BP-1-treated macrophage-conditioned medium enhances the proliferative activity of T-47D cells. T-47D cells were cultured with vehicle-MCM, MCM from macrophages treated with 10 μM BP-1, or vehicle-MCM supplemented with 10 μM BP-1. **(A,B)** Representative EdU staining images **(A)** and percentage of EdU-positive cells **(B)**. EdU-positive cells are shown in red, and nuclei were counterstained with DAPI (blue). **(C,D)** Representative colony formation images **(C)** and corresponding quantification **(D)**. Data are presented as the mean ± SD (n = 3). ns, not significant; *P < 0.05, **P < 0.01, and ***P < 0.001. BP-1, benzophenone-1; MCM, macrophage-conditioned medium: EdU, 5-ethynyl-2′-deoxyuridine.

## Glossary

*ABAT*: 4-aminobutyrate aminotransferase
*AKR1C1*: aldo-keto reductase family 1 member C1
*AKR1C3*: aldo-keto reductase family 1 member C3
AUC: area under the curve
BH: Benjamini–Hochberg
BP-1: benzophenone-1
*CA3*: carbonic anhydrase 3
CCK-8: Cell Counting Kit-8
*CD163*: cluster of differentiation 163
CD206: cluster of differentiation 206
*CD86*: cluster of differentiation 86
*CES1*: carboxylesterase 1
DEG: differentially expressed gene
EdU: 5-ethynyl-2′-deoxyuridine
ELISA: enzyme-linked immunosorbent assay
EMT: epithelial–mesenchymal transition
ER: estrogen receptor
FBS: fetal bovine serum
GEO: Gene Expression Omnibus
GO: Gene Ontology
GSEA: gene set enrichment analysis
*HSP90AA1*: heat shock protein 90 alpha family class A member 1
*HTRA1*: HtrA serine peptidase 1
IC_50_: half-maximal inhibitory concentration
IL-10: interleukin 10
KEGG: Kyoto Encyclopedia of Genes and Genomes
LASSO: least absolute shrinkage and selection operator
*LRP1*: LDL receptor related protein 1
*MARCKS*: myristoylated alanine-rich C-kinase substrate
MCM: macrophage-conditioned medium
NPT: ensemble with a constant number of particles, pressure, and temperature
NVT: ensemble with a constant number of particles, volume, and temperature
*OLFML3*: olfactomedin-like 3
PCA: principal component analysis
*PLAU*: plasminogen activator, urokinase
*PLAUR*: plasminogen activator, urokinase receptor
R^2^: scale-free topology fit index
Rg: radius of gyration
RMSD: root-mean-square deviation
RMSF: root-mean-square fluctuation
ROS: reactive oxygen species
ROC: receiver operating characteristic
SASA: solvent-accessible surface area
SCCS: Scientific Committee on Consumer Safety
SEA: Similarity Ensemble Approach
*SPHK1*: sphingosine kinase 1
SVM-RFE: support vector machine recursive feature elimination
*TGFBI*: transforming growth factor beta induced
*TIMP2*: TIMP metallopeptidase inhibitor 2
*TNF*: tumor necrosis factor
UMAP: uniform manifold approximation and projection
uPAR: urokinase-type plasminogen activator receptor (protein)
UV: ultraviolet
*VIM*: vimentin
WGCNA: weighted gene co-expression network analysis
XGBoost: extreme gradient boosting
