# Supplementary material for: Macrophage reprogramming by the ultraviolet filter benzophenone-1 promotes malignant phenotypes in breast cancer cells: integrated computational and experimental evidence
Source: Front Mol Biosci. 2026 Oct 13;13:1946836. doi: 10.3389/fmolb.2026.1946836 (PMC13710061; doi:10.3389/fmolb.2026.1946836)
Supplement: Supplementary file 1 [file Supplementaryfile1.pdf]

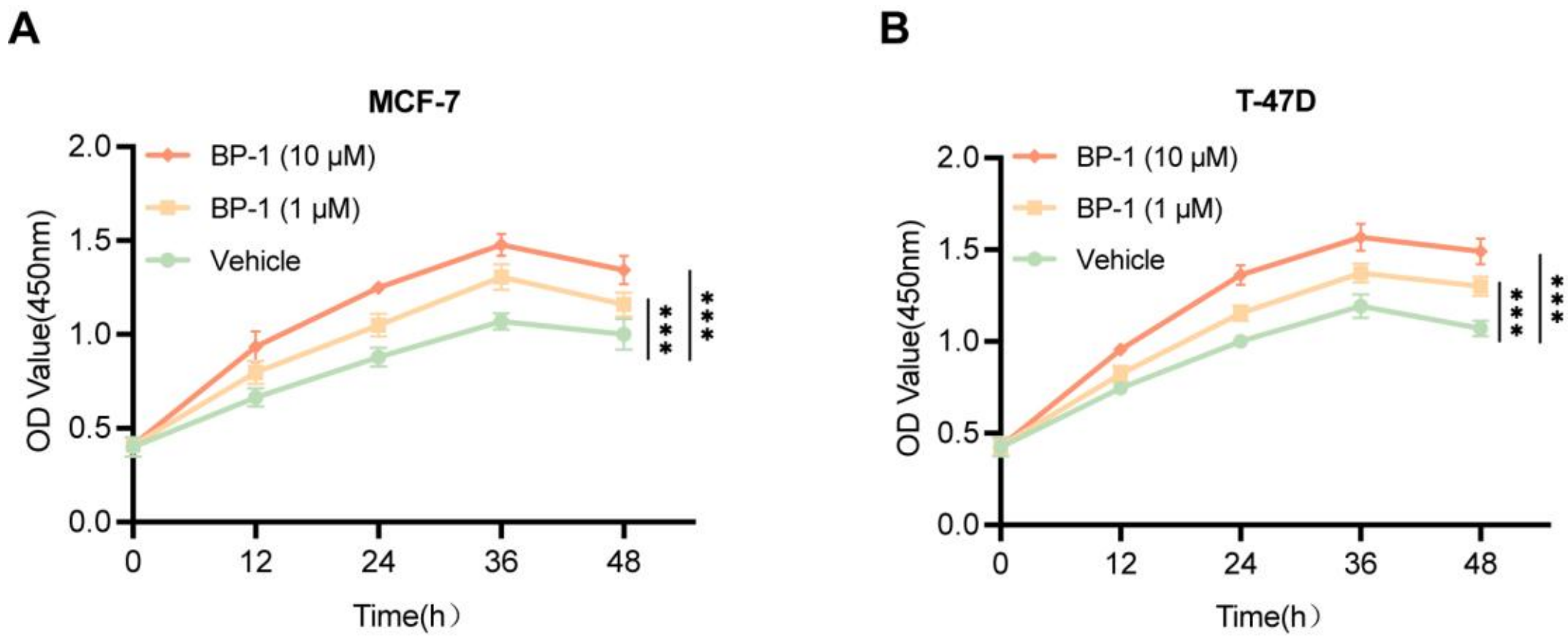

Supplementary Fig. S1. Effects of BP-1 on the viability of MCF-7 and T-47D cells. (A) MCF-7 cells and (B) T-47D cells were treated with vehicle or BP-1 (1 or 10 μM) for 0, 12, 24, 36, and 48 h. Data are presented as the mean ± SD ($n = 3$). ns, not significant; *$P < 0.05$, **$P < 0.01$, and ***$P < 0.001$. BP-1: benzophenone-1.

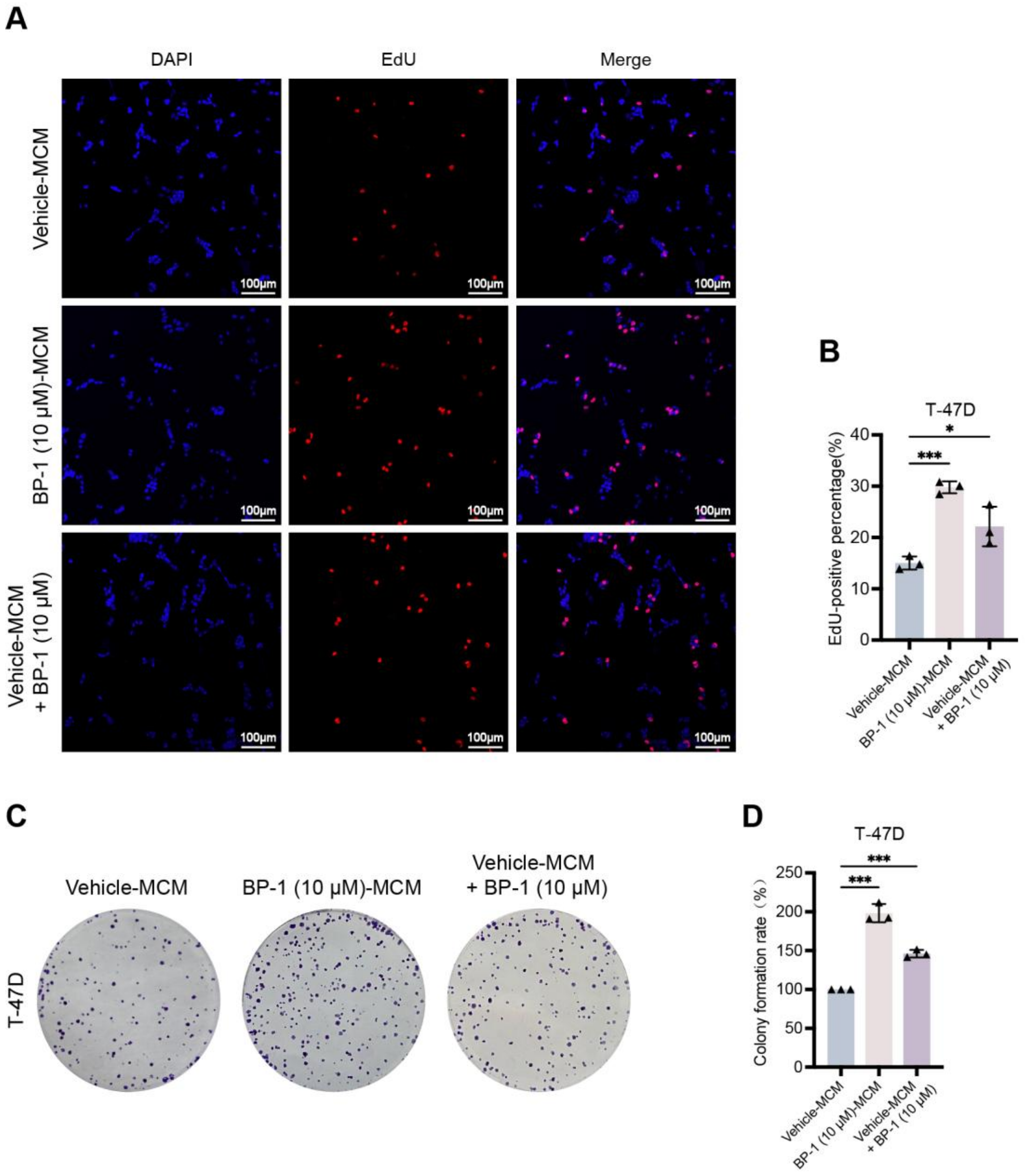

Supplementary Fig. S2. BP-1-treated macrophage-conditioned medium enhances the proliferative activity of T-47D cells. T-47D cells were cultured with vehicle-MCM, MCM from macrophages treated with 10 μM BP-1, or vehicle-MCM supplemented with 10 μM BP-1. (A, B) Representative EdU staining images (A) and percentage of EdU-positive cells (B). EdU-positive cells are shown in red, and nuclei were counterstained with DAPI (blue). (C, D) Representative colony formation images (C) and corresponding quantification (D). Data are presented as the mean ± SD (n = 3). ns, not significant; *P < 0.05, **P < 0.01, and ***P < 0.001. BP-1: benzophenone-1; MCM: macrophage-conditioned medium: EdU, 5-ethynyl-2′-deoxyuridine.
